# Cost‐effectiveness analysis of total intravenous vs. inhalation anaesthesia among adults aged ≥ 50 y undergoing major non‐cardiac surgery

**DOI:** 10.1111/anae.70288

**Published:** 2026-08-12

**Authors:** Samuel Frempong, Rebecca Kandiyali, James Mason, Louise Hiller, Janet Dunn, Katie Booth, Ramani Moonesinghe, Rupert Pearse, Ben Shelley, Shaman Jhanji, Joyce Yeung, the VITAL Trial Team

**Affiliations:** ^1^ Centre of Health Economics University of Warwick Coventry UK; ^2^ Warwick Clinical Trials Unit, Warwick Medical School University of Warwick Coventry UK; ^3^ Department of Targeted Intervention University College London London UK; ^4^ Faculty of Health & Medical Sciences University of Surrey Guildford UK; ^5^ School of Medicine, Dentistry & Nursing University of Glasgow Glasgow UK; ^6^ Department of Anaesthesia, Perioperative Medicine and Critical Care Royal Marsden NHS Foundation Trust London UK; ^7^ Division of Radiotherapy and Imaging The Institute of Cancer Research London UK

**Keywords:** cost‐effectiveness analysis, economic evaluation, general anaesthesia, inhalation anaesthesia, total intravenous anaesthesia

## Abstract

**Introduction:**

Total intravenous and inhalational anaesthesia are used widely to maintain general anaesthesia for major non‐cardiac surgery, yet their comparative cost‐effectiveness remains uncertain. The VITAL trial evaluated clinical outcomes, showing no difference in days alive and at home at 30 days. We conducted an economic evaluation alongside VITAL to determine whether total intravenous anaesthesia offers an economic advantage within the UK NHS.

**Methods:**

A within‐trial economic evaluation was conducted from the NHS and personal social services perspective over a 6‐month time horizon. Resource use was collected from trial records and questionnaires, and health‐related quality of life was measured using EuroQol five‐dimension five‐level instrument at baseline, discharge, 30 days and 6 months. Costs were evaluated using national sources and quality‐adjusted life years were calculated using the area under the curve approach. Incremental cost‐effectiveness ratios were estimated using imputed datasets, with uncertainty explored through bootstrapping and the probability of cost‐effectiveness illustrated using a cost‐effectiveness acceptability curve across a range of willingness‐to‐pay thresholds.

**Results:**

A total of 2507 patients were allocated randomly: 1253 (50%) received total intravenous anaesthesia; and 1254 (50%) inhalational anaesthesia. Mean costs and quality‐adjusted life years were similar across groups. Incremental cost was ‐£145 (95%CI ‐£1510–£1220) and incremental quality‐adjusted life years ‐0.001 (95%CI ‐0.008–0.005). Total intravenous anaesthesia showed a 56–57% probability of cost‐effectiveness at standard willingness‐to‐pay thresholds. Sensitivity analyses, including complete‐case and societal‐perspective models, yielded consistent findings of equivalence.

**Discussion:**

Total intravenous and inhalational anaesthesia show comparable cost‐effectiveness for adults aged ≥ 50 y undergoing major non‐cardiac surgery. Given clinical equipoise and equivalent economic outcomes, anaesthetic choice should continue to be guided by patient factors, clinician expertise and organisational context. Further research may be justified given the large population undergoing major surgery.

## Plain Language Summary

**What we did:**

Researchers compared two common ways of keeping people asleep during major surgery: total intravenous anaesthesia, which uses medicines given through a drip; and inhalational anaesthesia, which uses anaesthetic gases. They followed more than 2500 patients aged 50 years and older and measured both the costs of care and the patients' quality of life for six months after surgery.

**Why did we do it:**

Both types of anaesthesia are used widely, but it is not clear whether one provides better value for money. The researchers wanted to find out if one method costs less or leads to better recovery and quality of life for patients.

**What we found:**

The researchers found that the two types of anaesthesia performed very similarly. Patients had similar quality of life and recovery, and the costs to the NHS were almost the same. Neither approach showed a clear advantage over the other. This means that doctors can continue to choose the type of anaesthesia based on the patient's needs, their own experience and local hospital practices, rather than cost alone.

## Introduction

More than 1.5 million major non‐cardiac surgical procedures are performed annually in the UK NHS, and the delivery of high‐quality general anaesthesia is crucial for patient safety and positive surgical outcomes [1]. Within the NHS, general anaesthesia is maintained typically using inhalational volatile anaesthetic agents, such as sevoflurane or isoflurane [2]. However, total intravenous anaesthesia (TIVA), which involves the continuous infusion of propofol, has been used increasingly as an alternative technique for maintaining anaesthesia in recent years [3]. These two techniques exhibit distinct adverse‐effect profiles [4, 5]. Proponents of TIVA suggest that it offers safety comparable to inhalation anaesthesia, along with improved and faster patient recovery, including reduced postoperative nausea and vomiting, less acute pain, and a quicker return to consciousness [6, 7, 8]. Conversely, some are unconvinced of the superiority of TIVA in patient recovery and express concern regarding rare but serious risks, such as accidental awareness during surgery [9].

Currently, no large pragmatic trial in patients undergoing major non‐cardiac surgery has established definitively whether one technique is clinically or economically superior [10]. The VITAL trial is the largest pragmatic randomised evaluation of anaesthetic maintenance technique in this patient group undertaken to date in the UK [3]. It enrolled 2507 adults aged ≥ 50 y undergoing surgery across multiple specialities. The trial found no difference in days alive and at home at day 30 (DAH30), suggesting clinical equivalence between TIVA and inhalational anaesthesia [11].

Given this equivalence in clinical effectiveness, the economic dimension becomes central. If one technique is meaningfully less costly or yields better health‐related quality of life at similar cost, the NHS could consider promoting that technique more widely. Conversely, evidence of similar cost‐effectiveness would support the continuation of individualised, clinician‐driven practice.

The objective of this study was therefore to evaluate the cost‐effectiveness of TIVA compared with inhalational anaesthesia using robust trial‐based economic methods, aligned with the National Institute for Health and Care Excellence (NICE) guidance.

## Methods

A prospective within trial cost‐utility analysis was performed from a UK NHS and personal social services perspective using data from VITAL, a multicentre pragmatic randomised controlled trial conducted across UK hospitals. A 6‐month time horizon was chosen as it reflects the follow‐up period within the randomised controlled trial and captures most clinically relevant postoperative recovery and resource use in this population.

Adults aged ≥ 50 y undergoing elective major non‐cardiac surgery requiring general anaesthesia were eligible. Group allocation occurred before surgery. Exclusion criteria included contraindications to TIVA or volatile anaesthetic agents; emergency surgery; or inability to provide informed consent. Randomisation was stratified by surgical speciality; estimated duration of surgery; underlying pathology (cancer vs. non‐cancer); and frailty. The full protocol and trial design are described elsewhere [3].

Health resource use related to the index admission was captured via the Perioperative Quality Improvement Programme. This included details on the duration of anaesthesia; anaesthetic agents used for both induction and maintenance of anaesthesia; hospital duration of stay; and utilisation of critical care services. Follow‐up resource use (readmissions, outpatient visits, community care and social services) was collected at 6 months. For a broader societal perspective, additional questions considered lost productivity, such as work absence, during the six‐month period.

Valuation of resource use was conducted using health economic methods recommended by the NICE Guide to the Methods of Technology Appraisal [12]. Surgical procedure type influences costs and outcomes substantially, creating potential confounding when attributing differences between groups to the anaesthetic agent (the interventions being evaluated). To minimise potential confounding of outcomes within the trial design, randomisation was stratified by surgical specialty (musculoskeletal, intraabdominal, thoracic, vascular or other); expected duration of surgery (< 2 h or ≥ 2 h); underlying pathology (cancer or non‐cancer); and pre‐operative Rockwood Frailty Score (well, vulnerable or frail). Statistical analysis confirmed that the stratification variables were well balanced between treatment groups, thereby minimising confounding by surgical procedure. Consequently, the observed differences in costs and outcomes were assumed to be attributable to the anaesthetic agent used for maintenance of anaesthesia.

The economic analyses therefore aimed to value resource use attributable to the anaesthetic regimen used for maintaining anaesthesia. Costs were derived using the National Schedule of NHS Costs [13] and the Personal Social Services Research Unit compendium [14]. Anaesthetic drug costs were estimated using standard dosing assumptions where individual dose data were unavailable [15, 16]. Data on vapour flow rates for inhalational anaesthesia were also unavailable; therefore, costs were estimated assuming low‐flow techniques (0.5–1 l.min^‐1^), consistent with current NHS quality improvement initiatives promoting such practices for economic and environmental reasons [17, 18]. Costs are presented in 2022/23 £GBP and were inflation‐adjusted where necessary [18] (online Supporting Information Table S1). No discounting was applied due to the short time horizon.

Following NICE guidelines, the primary health outcome for the economic evaluation was the quality‐adjusted life year (QALY) [12]. The health‐related quality of life of patients was assessed at baseline, at hospital discharge, at 30 days and 6 months following randomisation using the EuroQol five‐dimension five‐level (EQ‐5D‐5L) instrument [19]. Responses to the EQ‐5D‐5L instrument were converted to utilities by mapping responses from the EQ‐5D‐5L to the EuroQol five‐dimension three‐level (EQ‐5D‐3L) valuation set using a mapping function [20] as recommended by NICE [11]. Quality‐adjusted life years were calculated for each patient as the area under the utility curve of the EQ‐5D utility scores from baseline to 6 months following randomisation using the trapezoidal rule [21].

The analysis followed intention‐to‐treat principles, with summaries and effect estimates calculated according to patients allocated trial groups (as per randomisation). Resource use categories, costs and utilities were summarised by trial group and between‐group differences were tested for statistical significance using Welch's two‐sample t‐test. A two‐sided significance level of 5% was used. Analyses were performed using Stata 16 (StataCorp, College Station, TX, USA) and results reported according to the Consolidated Health Economic Evaluation Reporting Standards checklist [22].

Missing costs and health utility data were imputed (the statistical process of replacing missing data with alternative plausible values) under the missing at random assumption (i.e. systematic causes of missingness are explained by predictor variables), at each time‐point using fully conditional multiple imputation by chain equations implemented through the MICE package in Stata 16. The missing at random assumption was assessed using logistic regression of cost and QALY missingness on baseline variables [23]. Imputation was performed by treatment group [23], generating 40 datasets using predictive mean matching (k‐nearest neighbour = 5) with relevant predictors. Costs and QALYs were analysed using seemingly unrelated regression adjusted for baseline variables, with estimates pooled using Rubin's rules [24]. Bootstrapping generated joint cost‐effect distributions and incremental estimates.

The cost‐effectiveness analysis is presented as an incremental cost‐effectiveness ratio, calculated as the ratio of mean differences in costs and QALYs between the interventions. Total intravenous anaesthesia was selected as the test intervention, while inhalation anaesthesia served as the control. For the base case model, 5000 bootstrapped replicates of incremental costs and QALYs were used to populate the incremental cost‐effectiveness ratio plane, reflecting sampling uncertainty. The net monetary benefit of TIVA was calculated using the bootstrap replicates evaluated at different willingness‐to‐pay thresholds, with a positive incremental net monetary benefit indicating cost‐effectiveness compared to inhalation anaesthesia. To illustrate the probability of cost‐effectiveness of TIVA across various willingness‐to‐pay thresholds, a cost‐effectiveness acceptability curve was generated from the net monetary benefit data. Further, the expected value of perfect information was computed from the net monetary benefit data and presented graphically. This metric quantifies the monetary value of eliminating all uncertainty from the cost‐effectiveness estimate across a range of willingness‐to‐pay thresholds, providing insight into the potential (upper‐bound) value of further research.

Sensitivity analyses were conducted to test the robustness of the cost‐effectiveness estimates. These analyses included estimating the cost‐effectiveness results using complete case data, adopting a wider societal perspective that incorporated productivity losses due to work absences, and re‐running the base‐case analysis using generalised structural equation modelling to assess alternative model specifications. This involved fitting a bivariate model in which costs were modelled using a gamma distribution with a log link, while QALYs were also modelled using the gamma family following transformation. Specifically, QALYs were transformed by subtracting each estimated QALY value from 0.5 (i.e. 0.5‐QALY) before modelling to ensure positivity, as none of the observed QALYs exceeded 0.5 and the gamma distribution requires strictly positive values.

## Results

A total of 2507 patients were allocated randomly: 1253 (50%) to TIVA and 1254 (50%) to inhalational anaesthesia. Baseline characteristics including age, sex, comorbidities, frailty and surgical specialty were well balanced between trial groups. There were 205 (8%) patients aged ≥ 80 y and 1381 (55%) were male. Surgical specialties included musculoskeletal, abdominal, vascular, thoracic and mixed procedures, reflecting broad generalisability.

Index admission costs were similar between TIVA and inhalational anaesthesia trial groups (Table 1 and online Supporting Information Table S2). Small differences in anaesthetic and post anaesthetic care unit level care costs reached statistical significance, but their absolute magnitude was small and unlikely to be clinically meaningful (see online Supporting Information Table S3). No statistically significant cost difference was observed for total NHS and societal costs among patients with complete data. The confidence interval included zero, indicating considerable uncertainty and no evidence of a true difference in costs. The EQ‐5D‐5L utilities were similar at all time‐points. Both groups showed the typical postoperative trajectory of initial decline followed by gradual recovery (Table 2).

**Table 1:** Mean NHS and social services costs by resource category between 0 and 6 months (complete cases only), in £GBP at 2022/2023 prices. Values are mean (SD).

	Total intravenous anaesthesia n = 1253	Inhalational anaesthesia n = 1254	Mean difference (95%CI)	p value
Resource use				
Anaesthesia	170 (83)	177 (80)	‐7 (‐13–0)	0.048
Ward/level 1 stay	10,948 (13,261)	10,683 (13,096)	265 (‐775–1305)	0.617
Level 2/HDU stay	1764 (4137)	1885 (4536)	‐121 (‐464–222)	0.490
Level 3/ICU stay	599 (5279)	551 (3578)	48 (‐308–404)	0.790
Enhanced care/PACU stay	228 (866)	327 (1361)	‐99 (‐189 to ‐8.9)	0.031
Index admission (total)	13,680 (17,979)	13,643 (16,856)	38 (‐1348–1424)	0.957
Readmissions	848 (2487)	943 (2530)	‐96 (‐310–119)	0.383
Outpatient visits	505 (969)	544 (1085)	‐39 (‐127–50)	0.393
Community care	229 (509)	226 (493)	3 (‐40–46)	0.897
Follow‐up to 6 months (total)	1571 (2692)	1666 (2809)	‐96 (‐356–165)	0.468
Work absence	3199 (7131)	3039 (6926)	160 (‐443–764)	0.603
Cost				
A: Cost (study intervention)	170 (83)	177 (80)	‐7 (‐13–0)	0.048
B: Cost (NHS contacts; excluding study intervention)	15,287 (19,196)	14,423 (12,869)	863 (‐684–2411)	0.274
C: Cost (work absence)	3199 (7131)	3039 (6926)	160 (‐443–764)	0.603
Cost (total (NHS) A + B)	15,468 (19,312)	14,632 (12,922)	836 (‐730–2403)	0.295
Cost (total (societal) A + B + C)	18,817 (21,483)	17,555 (14,134)	1261 (‐619–3142)	0.188

PACU, post anaesthetic care unit.

**Table 2:** EQ‐5D‐5L utility scores by time‐point and overall (area under the curve) and mean differences between groups (complete cases only). Values are mean (SD).

	Total intravenous anaesthesia n = 1253	Inhalational anaesthesia n = 1254	Mean difference (95%CI)	p value
EQ‐5D‐5L				
0 months	0.762 (0.241)	0.764 (0.246)	‐0.002 (‐0.021–0.017)	0.843
Discharge	0.669 (0.222)	0.678 (0.217)	‐0.008 (‐0.027–0.010)	0.388
30 days	0.737 (0.226)	0.743 (0.223)	‐0.006 (‐0.026–0.013)	0.509
6 months	0.755 (0.282)	0.762 (0.274)	‐0.007 (‐0.031–0.016)	0.546
QALY (area under the curve 0–6 months)	0.369 (0.101)	0.370 (0.096)	‐0.001 (‐0.010–0.008)	0.841

EQ‐5D‐5L, EuroQol five‐dimension five‐level; QALY, quality‐adjusted life years.

**Figure 1:**
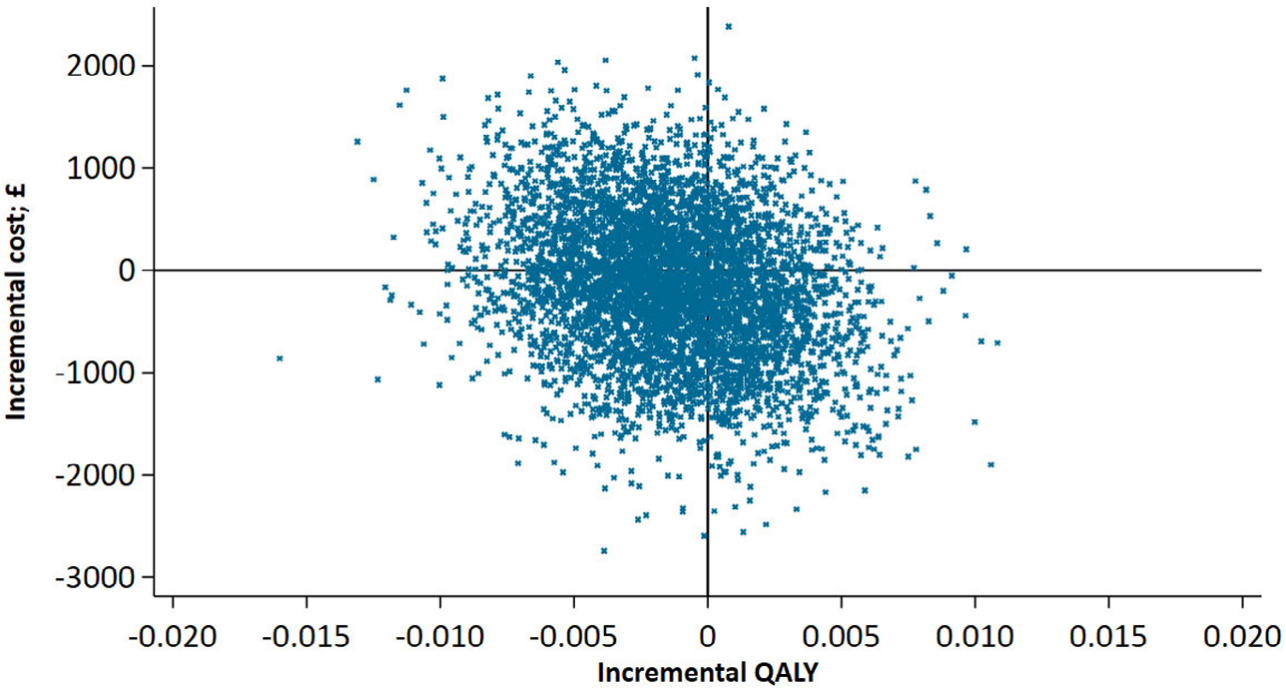
Cost–effectiveness plane showing the distribution of 5000 bootstrap replications of differences in costs (y‐axis) and effects (x‐axis), for total intravenous anaesthesia compared with inhalation anaesthesia. The quadrants indicate whether total intravenous anaesthesia is more/less effective and more/less costly than inhalational anaesthesia.

**Figure 2:**
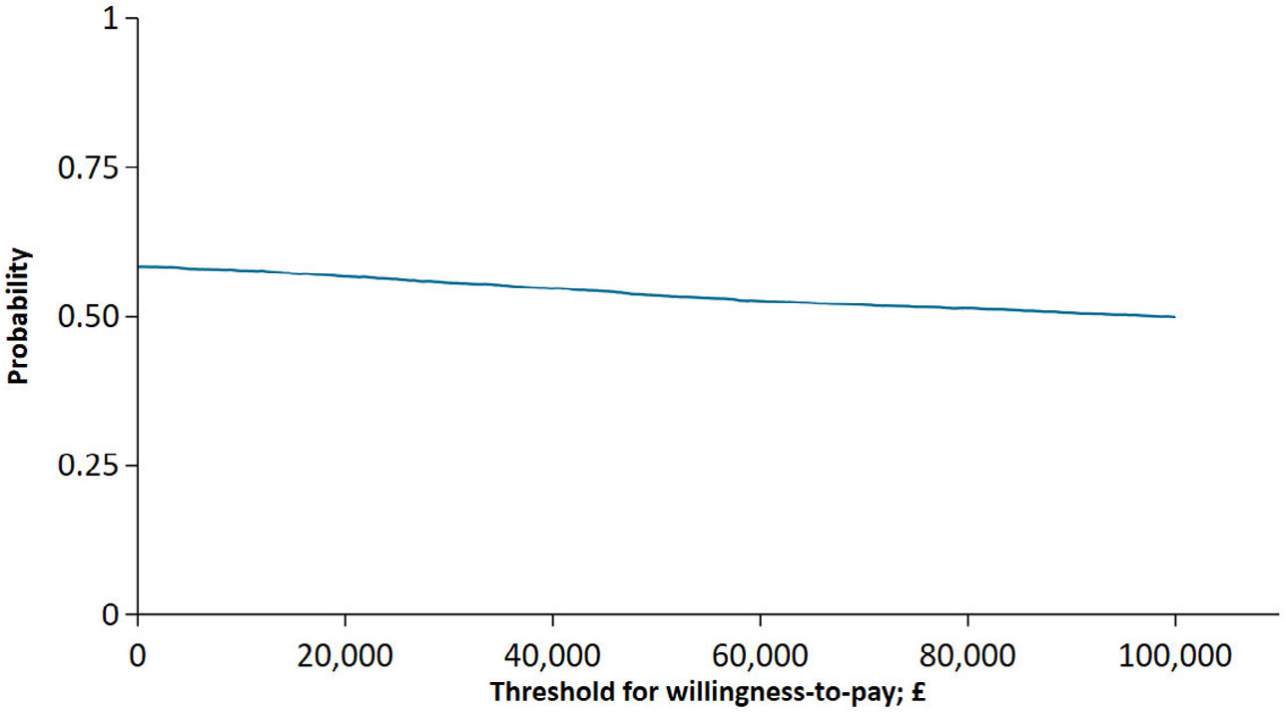
Cost‐effectiveness acceptability curve showing the probability that total intravenous anaesthesia is cost‐effective (y‐axis), depending on the willingness‐to‐pay per quality‐adjusted life year (x‐axis).

**Figure 3:**
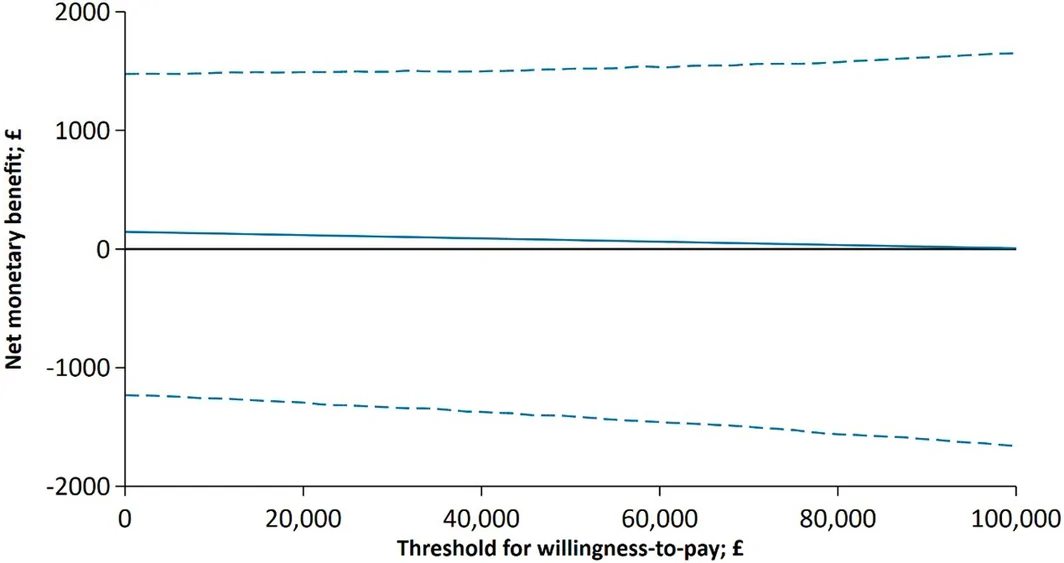
Net monetary benefit. This shows the estimated economic value of total intravenous anaesthesia (y‐axis) depending on the willingness‐to‐pay per quality‐adjusted life year (x‐axis), with dashed lines showing uncertainty around the estimates.

**Figure 4:**
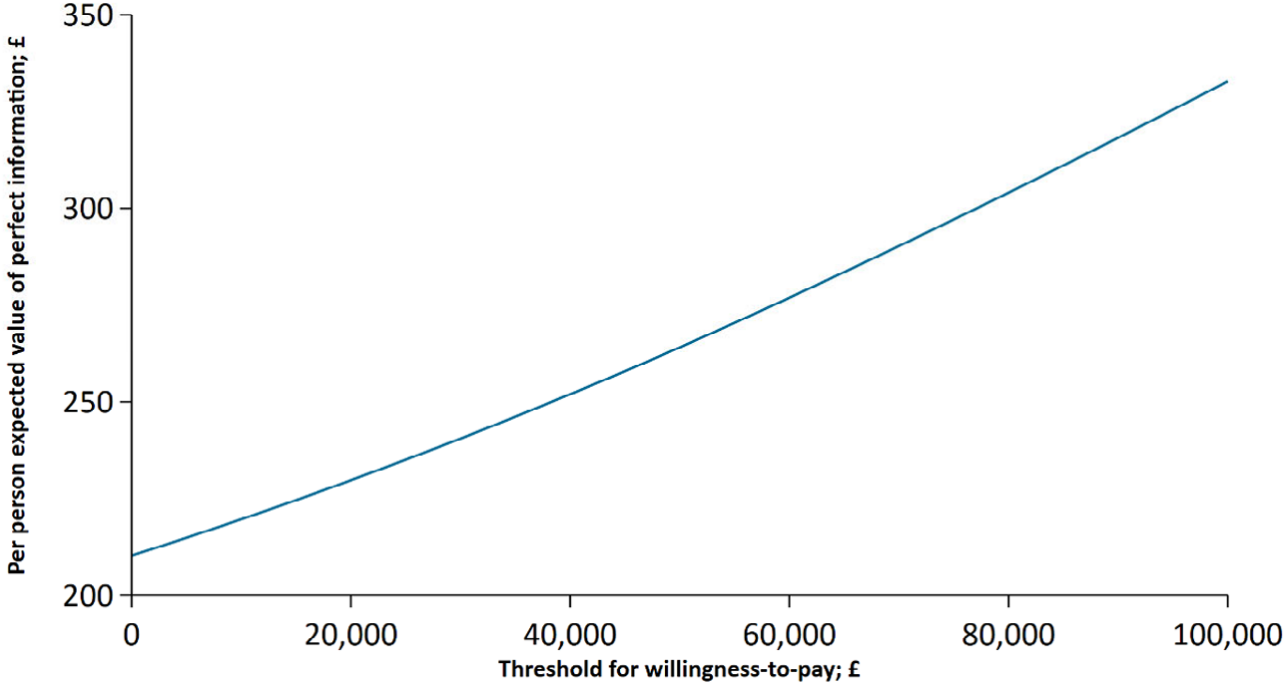
Expected value of perfect information. This shows the estimated value per patient of reducing uncertainty about whether total intravenous anaesthesia or inhalational anaesthesia is better (y‐axis), depending on the willingness‐to‐pay per quality‐adjusted life year (x‐axis).

Given minimal differences in costs and effects between total intravenous and volatile anaesthesia, the incremental cost‐effectiveness ratio was imprecisely estimated and not interpretable (Fig. 1 and Table 3). Total intravenous anaesthesia showed a 56–57% probability of cost‐effectiveness at standard willingness‐to‐pay thresholds (Fig. 2). Net monetary benefit estimates favoured TIVA marginally but with wide uncertainty intervals (Fig. 3). Per person expected value of perfect information values were < £250 (US$334, €290) (Fig. 4). Sensitivity analyses, including complete case, societal perspective and post‐hoc analyses, consistently supported the primary findings of no significant differences in cost and effectiveness.

**Table 3:** Cost‐effectiveness of total intravenous anaesthesia compared with inhalational anaesthesia (£GBP 2022).

Analysis	Incremental cost (95%CI)	Incremental QALYs (95%CI)	ICER	P^1^	P^2^	Net monetary benefit (95%CI)^1^	Net monetary benefit (95%CI)^2^	Expected value of perfect information^1^	Expected value of perfect information^2^
Base case									
Imputed costs and QALYs*	‐145 (‐1510–1220)	‐0.001 (‐0.008–0.005)	104,791	0.57	0.56	118 (‐1293–1490)	104 (‐1335–1495)	229.75	240.51
Sensitivity analyses									
Complete case costs and QALYs*	611 (‐824–2046)	‐0.002 (‐0.012–0.007)	Dominated	0.20	0.19	‐648 (‐2171–827)	‐666 (‐2223–822)	81.70	82.52
Societal perspective (imputed case)	7 (‐1457–1472)	‐0.001 (‐0.008–0.005)	Dominated	0.48	0.47	‐35 (‐1535–1442)	‐49 (‐1578–1458)	288.13	285.95
Post‐hoc analyses									
Model specification^3^	‐239 (‐1530–1052)	‐0.001 (‐0.008–0.006)	253,583	0.63	0.62	220 (‐1101–1536)	211 (‐1132–1565)	173.42	181.63

QALY, quality‐adjusted life years; ICER, incremental cost‐effectiveness ratio.

* Covariate adjusted.

^1^ Probability cost‐effective or net monetary benefit or expected value of perfect information if willingness to pay £20,000 per QALY.

^2^ Probability cost‐effective or net monetary benefit or expected value of perfect information if willingness to pay £30,000 per QALY.

^3^ Bivariate regression using generalised structural equation modelling framework; cost regression using Gamma family and log link, 0.5‐QALY transformation using gamma family.

## Discussion

This economic evaluation provides the largest and most rigorous comparison to date of the cost‐effectiveness of TIVA vs. inhalational anaesthesia for major non‐cardiac surgery within the NHS. The findings indicate that the two techniques are economically equivalent, with differences in costs and QALYs small, statistically non‐significant and clinically negligible. Combined with the VITAL trial's clinical findings, these results indicate that both techniques deliver comparable value within the NHS. The stability of findings across multiple sensitivity analyses reinforces their robustness. The implication is that anaesthetic choice should remain individualised, reflecting patient preference, clinician expertise and institutional factors rather than economic considerations alone.

Our headline result differs slightly from a recently published US model‐based analysis, which found TIVA to be the dominant strategy (less costly and more effective) [25]. However, the mean incremental within‐hospital cost differences in that study were only around £9 per patient (US$ 10, €8), which is small at the individual level, though could be relevant when extrapolated across larger populations. The apparent difference in that result was primarily driven by the more favourable adverse‐effect profile of TIVA regarding postoperative nausea and vomiting, which complements findings from our randomised controlled trial [3].

The choice of anaesthetic is a frequent decision, impacting a substantial number of patients. The patient‐level expected value of perfect information is extrapolated to the relevant population to inform the potential value of further research. To illustrate, assuming a willingness‐to‐pay of £20,000 (US$26,701, €23,165) per QALY and a target population of 1.5 million patients over a 5‐year technology time‐window, the population expected value of perfect information is estimated at £344 million (US$459 million, €398 million). This value represents the maximum potential value from resolving current decision uncertainty. The magnitude of the overall expected value of perfect information suggests that further research may be economically justifiable. However, the standard expected value of perfect information calculation and interpretation may require rethinking when the optimal decision may be to maintain both anaesthetic options rather than adopting a single preferred approach.

Because anaesthetic technique affects millions of operations annually, even small cost differences could theoretically accumulate to large system‐level effects. However, the uncertainty around cost estimates in this study means that any true difference is likely minimal. Population expected value of perfect information was substantial, reflecting the large population undergoing surgery, but its interpretation is nuanced given the practical need to maintain both techniques as part of clinical practice.

Strengths of this study include its large sample; pragmatic design; high follow‐up completeness; and alignment with contemporary NHS practice. The use of a randomised controlled trial design limits selection bias through random allocation, balances known and unknown confounders and reduces performance and detection biases in the comparison of the anaesthetic options. The trial design permitted the comprehensive assessment of health and social care resource use at follow‐up in the UK context. In addition, the within trial analysis design was validated by the convergence of differences in costs and outcomes within the trial follow‐up, removing the usefulness of exploring extrapolation of results to a longer time horizon (online Supporting Information Figure S1) and sensitivity analyses confirmed the robustness of our estimates.

Despite its strengths, several limitations warrant consideration. First, anaesthetic dosing for cost calculations relied on standard weight‐based estimations rather than individual patient data, although anaesthetic drugs represent only a small proportion of total peri‐operative costs. Second, environmental impact, including carbon footprint, was not included despite growing interest, particularly regarding volatile anaesthetics. Third, it is possible that the 6‐month time horizon may not capture longer‐term recovery differences, although extrapolation is unlikely to alter conclusions given the equivalent clinical trajectories observed. Finally, pragmatic trial design meant some aspects of care, such as analgesia or anti‐emetic use, were not protocolised and could vary between providers. However, this reflects real‐world practice and thus increases external validity.

Total intravenous and inhalational anaesthesia offer equivalent cost‐effectiveness for adults aged ≥ 50 y undergoing major non‐cardiac surgery. With no technique showing clear economic superiority, practice should continue to be guided by patient preference, clinician expertise and institutional resources. Future work examining environmental and long‐term economic implications may provide additional insights, but for now, both anaesthetic approaches remain equally justifiable within the NHS.

## Supporting information


**Figure S1.** Convergence plot showing the average differences in costs and health outcomes between groups over the trial.

**Table S1.** Unit costs of health and social care items and additional financial cost items.
**Table S2.** Completeness of data by follow‐up visit.
**Table S3.** Mean resource use by intervention group.
