# Supplementary material for: Cost‐effectiveness analysis of total intravenous vs. inhalation anaesthesia among adults aged ≥ 50 y undergoing major non‐cardiac surgery
Source: Anaesthesia. 2026 Aug 12;81(10):1378–85. doi: 10.1111/anae.70288 (PMC13551113; doi:10.1111/anae.70288)
Supplement: Supplementary file 1 [file ANAE-81-1378-s001.docx]

**Figure S1** Convergence plot. This shows the average differences in costs and health outcomes between groups over the trial.
