# Supplementary material for: Cost‐effectiveness analysis of total intravenous vs. inhalation anaesthesia among adults aged ≥ 50 y undergoing major non‐cardiac surgery
Source: Anaesthesia. 2026 Aug 12;81(10):1378–85. doi: 10.1111/anae.70288 (PMC13551113; doi:10.1111/anae.70288)
Supplement: Supplementary file 2 [file ANAE-81-1378-s002.docx]

**Table S1** Unit costs of health and social care items and additional financial cost items.

| Resource Item | Unit | Unit cost (GBP) | Source |
| --- | --- | --- | --- |
| Level of care |  |  |  |
| Ward/Level 1 | bed day | 1,698 | NHS Reference cost 2022-2023. Currency code XC07Z |
| Level 2/High dependency unit stay | bed day | 2,039 | NHS Reference cost 2022-2023. Currency code XC05Z |
| Level 3/Intensive care unit stay | bed day | 2,213 | NHS Reference cost 2022-2023. Currency code XC01Z |
| Enhanced care/PACU stay | bed day | 1,645 | NHS Reference cost 2022-2023. Currency code XC06Z |
| Readmissions |  |  |  |
| Non-Elective Inpatient - Long Stay | episode | 4,719 | PSSRU 2023, p. 37 |
| Non-Elective Inpatient - Short stay | episode | 857 | PSSRU 2023, p. 37 |
| Outpatients |  |  |  |
| Outpatient attendance | visit | 217 | PSSRU 2023, p. 37 |
| Emergency Medicine Service | visit | 190 | NHS Reference Cost 2022/2023.  Summary: OPD Attendances (Service code:180) |
| Community care |  |  |  |
| General practitioner (surgery) | 10-minute visit | 49 | PSSRU 2023, p. 65 |
| General practitioner (home visit) | 23-minute visit | 105 | PSSRU 2023, p. 65 |
| General practitioner (telephone contact) | contact | 9.40 | PSSRU 2023, p. 67 |
| General practitioner (online consultation) | episode | 43.92 | PSSRU 2023, p. 67 |
| Practice nurse | 15.5-minute visit | 12 | PSSRU 2023, p. 63 |
| District nurse | 20-minute visit | 19 | PSSRU 2023, p. 62 |
| Counsellor | 60-minute visit | 52 | PSSRU 2023, p. 57 |
| Speech and Language therapist | 60-minute visit | 52 | PSSRU 2023, p. 57 |
| Community physiotherapist | 60-minute visit | 52 | PSSRU 2023, p. 57 |
| Call to NHS 111 | contact | 13 | https://doi.org/10.3310/hsdr09210 |
| Call for ambulance/paramedic | incident | 244 | NHS Reference Cost 2022/2023. Ambulance sheet (Average of currency codes 01 – 04) |
| Mental health services | 60-minute visit | 56 | PSSRU 2015^a^, p. 199 |
| Meals on wheels | Per meal | 8 | PSSRU 2014^a^, p. 127 |
| Laundry | service | 19 | ONS, 2016^1^ |
| Personal social services |  |  |  |
| Social worker | 30-minute visit | 22 | PSSRU 2023, p. 76 |
| Care worker | 30-minute visit | 14 | PSSRU 2023, p. 79 |
| Family or patient support | 30-minute visit | 20 | PSSRU 2015^a^, p. 194 |
| Sitting services | 30-minute visit | 20 | PSSRU 2015^a^, p. 194  (Assumed to be same as support worker contact) |
| Medications |  |  |  |
| Propofol 200mg/20ml emulsion for injection | Vial | 5.28 | BNF^b^ |
| Propofol 1g/100ml emulsion for infusion | Vial | 26.40 | BNF^b^ |
| Sevoflurane 100% inhalation vapour liquid - 1 ml per 1 ml (250ml) | Bottle | 123 | BNF^b^ |
| Isoflurane 100% inhalation vapour liquid - 1 ml per 1 ml (250ml) | Bottle | 27.00 | BNF^b^ |
| Desflurane 100% inhalation vapour liquid - 1 ml per 1 ml (240ml) | Bottle | 120 | BNF^b^ |
| Neostigmine 2.5mg/1ml solution for injection | Ampoule | 1.5 | BNF^b^ |
| Sugammadex 500mg/5ml solution for injection | Vial | 149 | BNF^b^ |
| Dexamethasone (base) 3.3mg/1ml solution for injection | Ampoule | 2.4 | BNF^b^ |
| Dexmedetomidine 400micrograms/100ml infusion | Infusion bag | 24.64 | BNF^b^ |
| Entonox (nitrous oxide and oxygen) - (flogas rather than BOC) | Cylinder | 4.50 | NHSCC |
| Entonox disposable filter | Single | 0.71 | NHSSC REF: FTC127 |
| Remifentanil 1mg powder for solution for injection | Vial | 5.12 | BNF^b^ |
| Fentanyl 2.5mg/50ml solution for infusion | Vial | 9.8 | BNF^b^ |
| Alfentanil 1mg/2ml solution for injection | Ampoule | 0.9 | BNF^b^ |
| Midazolam 100mg/50ml solution for infusion | Vial | 9.96 | BNF^b^ |
| Ketamine 200mg/20ml solution for injection | Vial | 5.06 | BNF^b^ |
| Rocuronium bromide 100mg/10ml solution for injection | Ampoule | 5.7 | BNF^b^ |
| Morphine sulfate 50mg/50ml solution for infusion | Vial | 5.78 | BNF^b^ |
| Etomidate 20mg/10ml solution for injection | Ampoule | 1.38 | BNF^b^ |
| Vecuronium bromide 10mg powder for solution for injection | Vial | 11.7 | BP^C^ |
| Diamorphine 5mg powder for solution for injection | Ampoule | 2.56 | BNF^b^ |
| Oxycodone 10mg/1ml solution for injection | Ampoule | 1.6 | BNF^b^ |
| ^a^Unit cost has been inflated to 2022/23 prices  ^1^ONS,2016:<https://www.ons.gov.uk/economy/nationalaccounts/satelliteaccounts/compendium/householdsatelliteaccounts/2005to2014/chapter6homeproducedclothingandlaundryservices>  ^2^ONS,2022:<https://www.ons.gov.uk/employmentandlabourmarket/peopleinwork/employmentandemployeetypes/bulletins/averageweeklyearningsingreatbritain/september2022>  ^3^ONS,2022:<https://www.ons.gov.uk/employmentandlabourmarket/peopleinwork/employmentandemployeetypes/bulletins/averageweeklyearningsingreatbritain/september2022>  ^b^BNF: <https://bnf.nice.org.uk/drugs/>  ^C^British Pharmacopoeia: <https://www.pharmacopoeia.com/shop/products/1000021874?journey-type=CRS> | | | |

**Table S2** Completeness of data by follow-up visit. Values are number (proportion).

| Variable | TIVA, N = 1,253*^1^* | Inh, N = 1,254*^1^* | Overall, N = 2,507*^1^* |
| --- | --- | --- | --- |
| Health status | | | |
| EQ-5D-0 months | 1,207 (96) | 1,212 (97) | 2,419 (96) |
| EQ-5D-discharge | 1,068 (85) | 1,069 (85) | 2,137 (85) |
| EQ-5D-30 days | 1,061 (85) | 1,064 (85) | 2,125 (85) |
| EQ-5D-6 months | 1,055 (84) | 1,057 (84) | 2,112 (84) |
| EQ-5D- All visits | 841 (67) | 843 (67) | 1,684 (67) |
| Resource use | | | |
| Readmission_6_months | 1,047 (84) | 1,050 (84) | 2,097 (84) |
| Outpatient_6_months | 1,031 (82) | 1,036 (83) | 2,067 (82) |
| Community_6_months | 1,041 (83) | 1,039 (83) | 2,080 (83) |
| Work Absence_6_months | 1,053 (84) | 1,034 (82) | 2,087 (83) |
| *^1^* N (% not missing) | | | |

**Table S3** Mean resource use by intervention group. Values are mean (SD).

| Variable | TIVA, N = 1,253*^1^* | Inh, N = 1,254*^1^* | Difference*^2^* | p-value*^2^* |
| --- | --- | --- | --- | --- |
| Length of stay Ward/LEVEL1 (days) | 6.45 (7.81) | 6.29 (7.71) | 0.16 | 0.617 |
| Average length of stay LEVEL2 (days) | 2.09 (2.72) | 2.20 (2.99) | -0.10 | 0.558 |
| Average length of stay LEVEL3 (days) | 0.65 (3.67) | 0.59 (2.45) | 0.06 | 0.746 |
| Average length of stay PACU (days) | 0.34 (0.78) | 0.47 (1.22) | -0.14 | 0.031 |
| Average outpatient visits | 2.42 (4.46) | 2.59 (5.00) | -0.17 | 0.405 |
| Average GP surgery visits | 1.17 (2.05) | 1.19 (2.16) | -0.02 | 0.818 |
| Average GP home visits | 0.04 (0.29) | 0.03 (0.27) | 0.00 | 0.751 |
| Average GP Online consultations | 0.27 (1.28) | 0.40 (1.79) | -0.13 | 0.063 |
| Average Practice Nurse contacts | 1.06 (3.53) | 1.36 (5.72) | -0.30 | 0.146 |
| Average District Nurse contacts | 1.52 (7.78) | 1.99 (10.06) | -0.46 | 0.238 |
| Average Counsellor contacts | 0.16 (1.37) | 0.12 (0.92) | 0.04 | 0.410 |
| Average Speech and Language Therapist contacts | 0.01 (0.19) | 0.03 (0.65) | -0.02 | 0.339 |
| Average community Physiotherapists contacts | 0.16 (0.78) | 0.17 (0.91) | -0.02 | 0.609 |
| Average calls to NHS 111 (number per patient) | 0.10 (0.46) | 0.10 (0.42) | 0.00 | 0.917 |
| Average calls for Ambulance (number per patient) | 0.07 (0.32) | 0.08 (0.39) | -0.01 | 0.6596 |
| Average mental health services contacts | 0.04 (0.39) | 0.09 (1.00) | -0.05 | 0.129 |
| Average food at home services | 0.03 (0.87) | 0.25 (5.76) | -0.22 | 0.219 |
| Average Laundry Services used | 0.04 (0.90) | 0.17 (5.61) | -0.13 | 0.464 |
| Average social worker contacts | 0.04 (0.88) | 0.02 (0.25) | 0.02 | 0.500 |
| Average care worker contacts | 0.92 (13.21) | 0.87 (11.05) | 0.06 | 0.915 |
| Average Family Support contacts | 2.68 (18.87) | 1.30 (12.32) | 1.4 | 0.047 |
| Average sitting services | 0.01 (0.24) | 0.01 (0.19) | 0.01 | 0.543 |
| Average days off work | 30.69 (59.95) | 33.00 (62.10) | -2.3 | 0.403 |
| *^1^* Mean (SD)  *^2^* Welch Two Sample t-test  *^3^* CI = Confidence Interval | | | | |
